# Long-Term Clinical Outcomes in Patients with Chronic Rhinosinusitis with Nasal Polyps Associated with Expanded Types of Endoscopic Sinus Surgery

**DOI:** 10.3390/jcm13030866

**Published:** 2024-02-01

**Authors:** Daniel Martin-Jimenez, Ramon Moreno-Luna, Amparo Callejon-Leblic, Alfonso del Cuvillo, Charles S. Ebert, Juan Maza-Solano, Jaime Gonzalez-Garcia, Pedro Infante-Cossio, Serafin Sanchez-Gomez

**Affiliations:** 1 Rhinology Unit, Department of Otolaryngology, Head and Neck Surgery, Virgen Macarena University Hospital, 41009 Seville, Spain; mcallejon@us.es (A.C.-L.); jmanuel.maza.sspa@juntadeandalucia.es (J.M.-S.); jaime.gonzalez.sspa@juntadeandalucia.es (J.G.-G.); serafin.sanchez.sspa@juntadeandalucia.es (S.S.-G.); 2 Biomedical Engineering Group, University of Seville, 41004 Seville, Spain; 3 Rhinology and Asthma Unit, Department of Otolaryngology, Jerez University Hospital, 11407 Jerez, Spain; dr.cuvillo@comcadiz.es; 4 Department of Otolaryngology—Head & Neck Surgery, University of North Carolina School of Medicine, Chapel Hill, NC 27599, USA; charles_ebert@med.unc.edu; 5 Department of Surgery, School of Medicine, University of Seville, 41009 Seville, Spain; pinfante@us.es

**Keywords:** chronic rhinosinusitis, ethmoid, nasal polyps, nasal surgical procedures, paranasal sinuses, quality of life, SNOT-22

## Abstract

(1) Background: Surgical criteria for chronic rhinosinusitis with nasal polyps (CRSwNP) remain unresolved. This study addresses these discrepancies by comparing the clinical outcomes of expanded–functional endoscopic sinus surgeries (E–FESS) with more-limited FESS (L-FESS). (2) Methods: A database was analyzed retrospectively to compare surgical outcomes in CRSwNP patients who underwent E-FESS versus those subjected to L-FESS. Quality of life, endoscopic and radiological outcomes were compared at the baseline and two years after surgery. The clinical status of the responder was defined when a minimal clinically important difference of 12 points in SNOT-22 change was achieved. (3) Results: A total of 274 patients met the inclusion criteria and were analyzed; 111 underwent E-FESS and 163 were subjected to L-FESS. Both groups exhibited significant clinical improvements, although a greater magnitude of change in SNOT-22 (14.8 ± 4.8, *p* = 0.002) was shown after E-FESS. Higher significant improvements for endoscopic and radiological scores and lower surgical revision rates were also noted in the E-FESS group. (4) Conclusions: E-FESS provides better clinical outcomes and reduced revision surgery rates when compared to L-FESS in CRSwNP patients two years after surgery, irrespective of any comorbidity. Further randomized prospective studies are needed to comprehensively contrast these results.

## 1. Introduction

Chronic rhinosinusitis with nasal polyps (CRSwNP), an inflammatory sinonasal disease affecting between 0.5–4.5% of the general population, poses not only a significant impact on patients’ quality of life (QoL) but also a high economic burden on healthcare systems [1,2]. Recent advances in the understanding of the underlying inflammatory processes at a molecular level have led the way for new precision medicine paradigms to be applied and for new therapies aimed at controlling the inflammatory cascade to be developed [3,4,5]. Despite the demonstrated effectiveness of medical treatment with intranasal steroids and intermittent cycles of systemic corticosteroids, endoscopic sinus surgery (ESS) continues to serve as a suitable alternative in those patients whose symptoms do not positively respond to appropriate medical treatment [6].

The introduction of inflammatory endotypes and biomarkers aimed at classifying patients has considerably changed standard surgical procedures [7,8], especially in patients with type 2 (T2) inflammatory profiles [9,10]. However, to date, a lack of consensus regarding the optimal surgical strategy remains apparent, with the recurrence of polyps being a frequent constraint in CRSwNP patients [11,12]. While limited functional ESS (L-FESS) were initially described with the aim of anatomically restoring mucociliary clearance [13,14,15], recent technical advancements have now enabled the development of expansive FESS (E-FESS), which involve the resection of bony sinus structures coupled with the treatment of the mucosa covering the sinonasal cavity [16,17]. These approaches are driven by novel theories that integrate anatomical and inflammatory concepts (i.e., the mucosal concept), aimed at enhancing comprehension and management of the CRSwNP disease [18]. Although E-FESS has often been described as a complete sphenoethmoidal resection, associated with a maxillary antrostomy and a modification of the frontal sinus ostium [19,20,21], different surgical definitions, with no clear criteria by which to comprehensively define their appropriate extent, coexist in the literature, rendering an effective comparison of results among studies especially difficult [22,23].

In this sense, some studies have already reported improved clinical outcomes and lower recurrence rates associated with E-FESS, even in patients with more severe and recalcitrant phenotypes. Similarly, more extended approaches to the frontal sinus (i.e., frontal sinusotomy type Draf IIb-III) and the medial wall of the maxillary sinus are beginning to be redefined for the more aggressive forms of CRSwNP associated with T2 inflammatory features [24,25]. However, limitations such as small sample sizes, short follow-up periods, an inaccurate characterization of phenotypes and an inconsistent adoption of standardized approaches persist, which undermine the available scientific evidence of surgical outcomes in CRSwNP patients [26].

A preliminary publication by our research group has already provided an initial exploration of QoL outcomes [27]. Thus, the main objective of this study was to comprehensively analyze clinical outcomes in CRSwNP patients subjected to E-FESS compared with others subjected to L-FESS over a follow-up period of two years. This investigation seeks to furnish supplementary insights not only into QoL metrics but also encompassing endoscopic and radiological measurements. It is hoped that this study will strengthen the available evidence on E-FESS outcomes and help provide new surgical recommendations in CRSwNP patients.

## 2. Materials and Methods

### 2.1. Design and Participants

A non-randomized interventional retrospective study from a database of CRSwNP patients who had undergone ESS in the rhinology unit of a tertiary-care level hospital from January 2016 to March 2023 was performed. The sample was divided into two surgical groups: (i) a first subgroup of patients who had undergone E-FESS; and (ii) a second subgroup subjected to L-FESS. Data were systematically collected from patients’ medical records, before and two years after surgery. Inclusion criteria were patients older than 18 with a diagnosis of CRSwNP based on the EPOS 2020 criteria [1] and a polyp size greater than or equal to 2 (at least 1 in each nostril) on the nasal polyp score (NPS) [28] and with a moderate-to-severe self-reported impact (≥20 points) on the Sinonasal Outcomes Test-22 (SNOT-22) [29]. Excluded from the study were women who were either pregnant or breastfeeding, patients with eosinophilic granulomatosis with polyangiitis or severe systemic diseases (excluding bronchial asthma or nonsteroidal anti-inflammatory drug (NSAIDs-exacerbated respiratory disease (N-ERD)), neoplasms or pathologies related to the abuse of vasoconstrictor agents (e.g., oxymetazoline), patients with unilateral nasal inflammatory disease or without nasal polyps and those in treatment with monoclonal antibodies during the period of the study. All patients fulfilled the surgical criteria according to RAND/UCLA methodologist to undergo surgery for CRSwNP [30].

### 2.2. Surgical Technique and Postoperative Cares

The type of surgery and its extension (E-FESS or L-FESS) were dictated by the intraoperative clinical judgment of the enrolling expert rhinologists according to individual disease state. An extended surgery was prescribed in CRSwNP patients with poor prognostic comorbidities (e.g., uncontrolled asthma, severe N-ERD and a history of ≥2 previous ESS), as well as a partial/total opacity of all sinuses and blockage of the osteomeatal complex (i.e., CT-scan Lund–Mackay scale ≥14 points). By contrast, L-FESS was conducted on patients with no or well-controlled comorbidities and/or partial sinus CT opacities noted on imaging. Such criteria have been commonly employed in previous studies [16,19,25,31]. All developed surgeries included a bilateral approach.

**Figure 1:**
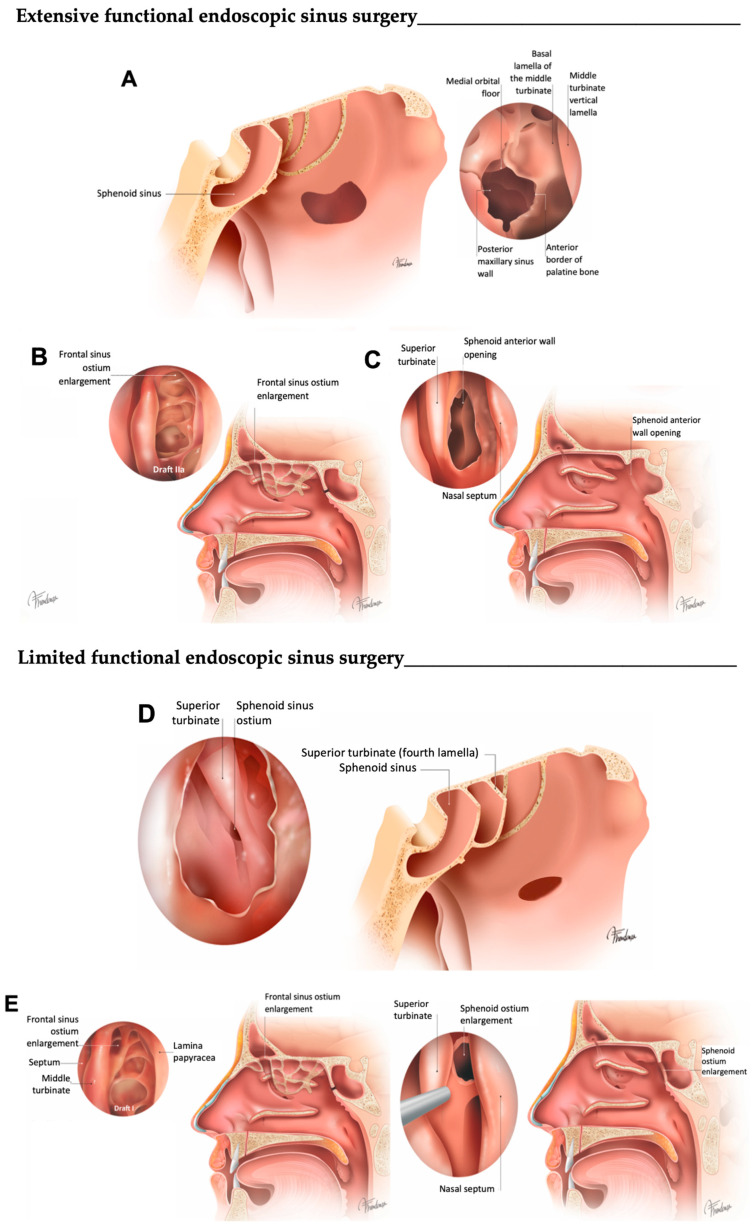
Graphical definition of E-FESS and L-FESS. (**A**) A complete resection of the anterior and posterior ethmoidal cells, opening the four lamellas, are performed, leading to a wide exposure of the anterior skull base, the posterior frontal sinus table and the lamina papyracea, and a maxillary antrostomy is associated. Middle meatal antrostomy is performed as widely as possible. (**B**) Frontal sinus is opened to expose and modify the ostium (Draf IIA frontal sinusotomy or superior). (**C**) Sphenoidal ostium is opened and modified until a complete exposure of planum. (**D**) A partial resection of the bony lamellas is performed, without completely exposing the anterior base of the skull (i.e., the bony septa are consecutively opened until the sphenoidal ostium is accessed transethmoidally). The opening of the maxillary ostium is conducted on demand, with an attitude as conservative as possible. (**E**) Frontal sinus is opened conservatively, without modifying the sinus ostium, and the sphenoid sinus ostium is enlarged.

E-FESS technique consisted of a full resection of the anterior and posterior ethmoidal cells as well as a sphenoidotomy and maxillary antrostomy. The frontal sinusotomy consisted of at least a Draf IIa. Subsequently, the residual septa were completely removed, leading to a wide exposure of the anterior skull base, the posterior frontal sinus table and the lamina papyracea. More conservative approaches targeting osteomeatal complex disease to allow for proper sinus ventilation, mucociliary clearance and easy topical therapy instillation were operationalized as L-FESS (as seen in Figure 1). Detailed features of both surgical approaches are outlined in Table 1.

Postoperative care was the same for both surgical groups: the removal of the non-absorbable nasal packing 48 h after surgery and gentle nasal lavage with seawater or physiological saline three times a day. In cases of associated endoscopic septoplasty, silicone sheets to protect the septum mucosa and avoid synechia for at least three weeks after surgery were used. Nasal rinses and intranasal corticosteroids were reintroduced three weeks after surgery in both surgical groups.

### 2.3. Data and Outcome Measures

Demographic and clinical variables were collected. These data include age, gender, a previous history of smoking, asthma, N-ERD, previous ESS, allergic sensitization (atopy) and use of systemic corticosteroids (SCS). Presurgery serology, including total immunoglobulin (Ig) E levels and peripheral eosinophils counts, were also collected.

QoL was assessed at the baseline and two years after surgery using the SNOT-22 questionnaire, with each item rated on a 6-point scale (0 = no problem; 5 = most serious problem) and final outcome scores reporting the severity of symptoms in the range of 0–110 [32,33]. Endoscopic and radiological measures were also considered. Endoscopic variables were assessed using the NPS rating from 0 to 4 for each nostril separately and based on the visualization of the extent and volume of the nasal polyp in the nasal cavity [28], and the modified Lund–Kennedy (MLK) scoring system from 0 to 12, where higher scores represent worse bilateral disease severity (polyps, oedema and nasal discharge) [34]. Baseline polyps scores were systematically recorded during the preoperative visit and later confirmed in the operation room before surgical intervention. Radiological impairment (non-contracted CT-scan of paranasal sinuses) was measured by the Lund–Mackay (LM) scale, ranging from 0 (complete lucence of all sinuses) to 24 (complete opacity of all sinuses) [35]. Endoscopic and radiological measurements were recorded and reviewed at follow-up by the same expert rhinologists. Discrepancies were solved by consensus. During the follow-up period after surgery, major complications, such as cerebrospinal fluid leaks, severe hemorrhage, orbital complications or toxic shock syndrome, were also recorded.

### 2.4. Statistical Analysis

Descriptive statistics for patient demographics and baseline characteristics were performed. Pearson’s chi-square (χ^2^) test was used to assess differences in the prevalence of categorical variables in the two groups, and a *t*-test was used to analyze differences in outcome measures. The normality of variables was evaluated using the Kolmogorov–Smirnov test. Differences in SNOT-22 scores between baseline and the follow-up period in the same group and between groups were evaluated through unpaired and paired *t*-tests, respectively. To assess the association of QoL outcomes with the type of surgery performed, simple linear regression models were carried out. The main exposure variable of interest was the surgical group (E-FESS versus L-FESS), whereas the main outcome of interest (dependent variable) was the changes in SNOT-22 scores (SNOT-22 (preoperative)–SNOT-22 (postoperative)). Multiple linear regression was performed to assess potential confounding effect estimates due to covariates such as gender, age, smoking, asthma, N-ERD, previous ESS, blood eosinophilia, peripheral blood IgE, atopy and cycles of SCS. Baseline NPS, MLK scale, LM range and SNOT-22 scores were also evaluated. Thereafter, simple and multiple linear stepwise regression analyses (backward and forward) were also performed with the aim of obtaining more robust statistical results. Models were adjusted following the Bayesian information criterion (BIC) and results were reported using unadjusted and adjusted effect estimates (β), standard error (SE), 95% confidence intervals (CI) and corresponding *p*-values. The coefficient of multiple determination values (R2) was used to assess model fitting, and variance inflation factors (VIFs) were calculated to quantify multicollinearity.

Subsequent to the follow-up period, assessments of endoscopic outcomes (employing NPS and MLK scale), as well as radiological evaluations (LM score), were conducted. The mean changes for these same variables were analyzed through a paired *t*-test, and comparisons between groups were performed via an unpaired *t*-test. Simple and multiple linear stepwise regression analyses (backward and forward) were also performed. In these models, the same criteria for information and communication of results used in the previous regression calculations were followed.

Revision surgery rate and major complications after surgery were also compared using χ^2^ test. A *p*-value of 0.05 was considered significant in our analysis. Data analysis was performed using the IBM SPSS Statistics 28.0 package for Windows (New Orchard Road Armonk, NY, USA).

## 3. Results

A total of 274 patients were included in the study; 111 (40.5%) underwent E-FESS and 163 L-FESS (59.5%). Supplementary Table S1 shows the distribution of variables such as gender, age, asthma, N-ERD, previous ESS, blood biomarkers and allergy. Endoscopic, radiological and QoL are also shown for both surgical groups at the baseline. There were differences in the frequency of previous ESS between groups, with more frequent interventions (40.5%) in the group subjected to E-FESS compared with the 27.6% of the L-FESS group (*p* = 0.025). Endoscopic and radiological scales also reflected higher baseline scores in the E-FESS group. No differences were detected for other variables of interest, such as asthma, N-ERD, blood biomarkers and baseline QoL scores.

### 3.1. QoL Outcomes

A significant decrease in SNOT-22 was observed in both surgical groups from baseline to the 2-year follow-up, i.e., from 68.1 ± 21.2 to 28.9 ± 22.5 (*p* < 0.001) in the E-FESS group and from 62.1 ± 23.0 to 34.5 ± 28.4 (*p* < 0.001) in the L-FESS group. In addition, significant differences between groups in SNOT-22 change were observed, with greater improvement in the group of patients who underwent E-FESS (*p* = 0.028).

A multiple linear regression model showed that patients who underwent E-FESS achieved on average an improvement in SNOT-22 of 12.2 points (β = 12.2 ± 5.0, 95% CI: [2.3, 22.0], *p* = 0.016) higher than those who received L-FESS. Additionally, patients with higher baseline SNOT-22 scores also obtained greater improvement (β = 0.7 ± 0.1, 95% CI: [0.4, 0.9], *p* < 0.001) (see Table 2 and the equation below in the caption). Although, in the simple linear regression model, higher baseline NPS scores were associated with greater improvement in SNOT-22 change (β = 2.8 ± 1.1, 95% CI: [0.7, 5.0], *p* = 0.009), such an effect was no longer seen after adjusting for covariates in the multiple regression analysis. MLK scale, Lund–Mackay scores and the other variables analyzed were not significantly associated with SNOT-22 change.

A supplementary multiple linear model was employed by performing stepwise backward–forward regression. The model with the lowest BIC (see Table 3) was selected. This model confirmed that patients who underwent E-FESS (β = 14.8 ± 4.8, 95% CI: [5.5, 24.1], *p* = 0.002) and had higher baseline SNOT-22 scores experienced greater improvement at the follow-up (β = 0.6 ± 0.1, 95% CI: [0.4, 0.8], *p* < 0.001). Interestingly, this model also showed that a previous history of ESS reduced the improvement to 11.2 points (β = −11.2 ± 5.0, 95% CI: [−21.2, −1.3], *p* = 0.027).

Supplementary Table S2 reports that a previously published multiple logistic regression model showed that subjects who underwent E-FESS, after adjusting for covariates, exhibited a 6.5-times-higher odds ratio of being a responder at the 2-year follow-up (OR = 6.5; 95% CI: [1.7, 24.8], *p* = 0.006); whereas a previous history of ESS had the opposite effect (OR = 0.2; 95% CI: [0.0, 0.7], *p* = 0.010). Noteworthy, patients who were treated with E-FESS had a ratio 2.9-times-higher odds of being a super-responder (OR = 2.9; 95% CI: [1.1, 7.8], *p* = 0.038), after adjusting for baseline SNOT-22 score (OR = 1.1; 95% CI: [1.0, 1.1], *p* < 0.001). Female gender and a history of previous ESS were also related to a reduction in the probability of reaching the minimal clinically important difference (MCID). Other variables such as asthma, N-ERD, atopy or blood test results were not found to be associated.

### 3.2. Endoscopic and Radiological Outcomes

**Figure 2:**
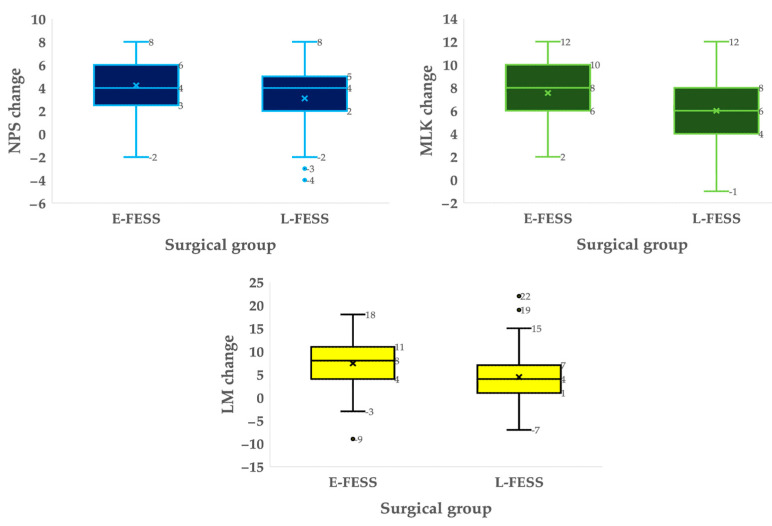
Boxplot distributions of nasal polyps score (NPS), modified Lund–Kennedy scale (MLK) and Lund–Mackay score (LM) changes between the two groups. Significant differences in endoscopic and radiological score changes between groups were detected using unpaired T–student (NPS: *p* < 0.001; MLK scale: *p* < 0.001; LM score: *p* < 0.001). Crosses indicate the mean value of change for each variable, while the horizontal lines correspond to the median.

Baseline and 2-years after surgery scores for the endoscopic and radiological scales are shown in Supplementary Table S3. A significant mean improvement was seen in NPS, with a mean change of 4.2 ± 2.3 points in the E-FESS group and 3.1 ± 2.6 points in limited surgeries (E-FESS group: from 8.6 ± 2.0 to 4.1 ± 3.3, *p* < 0.001; L-FESS group: from 7.6 ± 2.2 to 4.4 ± 3.6, *p* < 0.001). Similar outcomes were observed in MLK scores (*p* < 0.001) (Figure 2). Differences in postoperative endoscopic scores were also observed between groups in NPS (*p* = 0.037) but not in the MLK scale (*p* = 0.504). According to the LM scale change, a statistically significant difference was also obtained (E-FESS group: from 16.8 ± 5.1 to 9.6 ± 4.7, *p* < 0.001; L-FESS group: from 15.0 ± 5.6 to 11.6 ± 6.3, *p* < 0.001), as with postoperative measurements (*p* = 0.018) (Figure 2).

A multivariate linear model was derived by performing stepwise backward–forward regression with asthma, N-ERD, previous ESS and type of surgery, as well as with sex and age being considered as independent predictors. The model with the lowest BIC (see Table 4 and the equation below) was selected as the best model. In this model, subjects with a higher baseline NPS value experienced, on average, a greater improvement in polyps size at follow-up (*p* < 0.001). Worst values in baseline SNOT-22 poorly reduced this improvement (*p* = 0.046). Similar outcomes were observed for MLK scale changes, with greater improvements in patients with higher baseline scores (*p* < 0.001). There were lower enhancements in endoscopy when greater opacification on baseline CT-scan was reported (*p* = 0.013). Additionally, Table 4 shows the multivariate linear model for the change in LM scale. A greater sinus opacification in the baseline CT-scan reported greater improvements in the radiological image at follow-up (*p* < 0.001).

### 3.3. Revision Surgery Rates and Complications

A significant statistical difference rate of reintervention in the E–FESS group (6.3%) compared to the L-FESS group (17.8%) was observed (*p* = 0.006). No differences were found between groups in terms of major postoperative complications (E-FESS group: 2.7%; L-FESS group: 3.7%; *p* = 0.079).

## 4. Discussion

This study included patients with moderate-to-severe CRSwNP divided into two surgical groups, E-FESS and L-FESS. There was an observed significant enhancement in clinical outcomes in both groups, but especially in the E-FESS group, in which patients exhibited a greater likelihood of achieving the responder status. Significantly superior improvements in endoscopic and radiological measurements, as well as reduced rates of revision surgery, were also reported for patients undergoing E-FESS.

### 4.1. QoL Outcomes

QoL, as measured by the SNOT-22 score, showed a significant improvement after surgery in both surgical groups. To better quantify the effects of the surgical treatment with respect to baseline [36,37], we analyzed SNOT-22 change at the 2-year follow-up; the results showed a greater mean improvement of 39.2 in patients who underwent E-FESS, versus 27.6 points in those who underwent L-FESS (*p* = 0.028), which corresponds to previous studies where similar surgical extent were compared [16,19,25,38]. However, there is heterogeneity in the results published in the literature, with reported SNOT-22 change values ranging from 12.7 to 44.8 points [15,32,39,40]. According to the present results, such a divergence in the magnitude of SNOT-22 change after surgery may be partly due to the different surgical approaches used in these studies, which show inherently different abilities to control local inflammation [9,10,16,19,25,41,42], and their extent.

In fact, both simple and multiple linear regression models conducted in our study showed that patients who were subjected to E-FESS achieved a higher improvement in QoL outcomes at the two-year follow-up independently of poor prognosis features such as asthma, N-ERD or blood biomarkers of T2 inflammation (Table 2). These findings differ from those of other authors who did find an association of these comorbidities with poorer postsurgical QoL outcomes and higher recurrence rates in asthma and N-ERD patients [25,41], as well as with elevated peripheral blood T2 phenotype biomarkers (i.e., eosinophilia, total IgE count) [20,42]. To reinforce whether such a differential gain was biased by baseline differences between groups or the presence of uncontrolled confounding factors, a more statistically robust multiple stepwise linear regression algorithm was performed. After adjusting by baseline SNOT-22 scores, the model confirmed a significantly higher mean SNOT-22 change of 14.8 in patients undergoing E-FESS (Table 3). The same effect of baseline SNOT-22 scores on postsurgical improvement has been previously highlighted in the literature, showing that patients with higher baselines scores are more likely to reach greater improvements after surgical intervention [43,44]. Additionally, a negative effect of a previous history of ESS was seen, which reinforces the poor prognosis tendency reported for revision surgeries in CRSwNP patients (Table 3) [10,12,45].

The MCID for SNOT-22 is a specific measure used to identify a patient’s improvement in QoL [36]. Multiple logistic regression models were performed, and previously published [27], using the MCID as dependent variable. In these analyses, surgical extent and previous history of ESS were the only variables influencing the probability of becoming a responder at the 2-year follow-up (Supplementary Table S2). Similar outcomes were observed when further multiple stepwise logistic regression models were used to identify the features that most influenced the probability of becoming a super-responder (i.e., achieving an improvement twice the MCID). The model with lower BIC showed that the type of surgery remained the most important feature (Supplementary Table S2). Therefore, the previously published results, which are supplemented in this study, reinforce the main role of the surgical approach and its extent in QoL outcomes [10,16,19,25,41], as well as its influence on the definition of new clinical concepts (responder and super-responder) in CRSwNP.

Although further knowledge has been gained during the last few years regarding the inflammatory cues in CRSwNP [7,46], T2 inflammatory biomarkers and comorbidities still have an uncertain role in the management of these patients. For this reason, further criteria with which to clinically characterize and phenotype CRSwNP patients are still needed [47]. In previously published results, which are included in Supplementary Table S2, baseline variables representative of T2 inflammation did not show any significant association with the probability of achieving MCID after surgery. Therefore, we emphasize that the extent of surgery may play a pivotal role in the surgical management of CRSwNP patients, even in those with more severe and recalcitrant phenotypes [27].

### 4.2. Endoscopic and Radiological Outcomes

As anticipated in endoscopic assessments, a noteworthy enhancement in polyp size was noted, exhibiting a greater mean improvement among patients subjected to E-FESS (Figure 2). These findings align with previously documented studies reporting diminished average NPS values following more expanded FESS [10,38,48], as opposed to inferior outcomes observed after limited surgical interventions [15,25]. Curiously, although significant improvements in scores were also observed in the MLK scale in both surgical groups, significant differences were found in the long-term scores at baseline (Figure 2) but not at the end of monitoring (*p* = 0.591). These outcomes closely resembled those reported by other authors employing various extended approaches [17,19]. Following the execution of multiple linear regression models, it was observed that changes after surgery in the NPS and MLK scales were predominantly influenced by the baseline values of both endoscopic scales. Elevated baseline values exhibited a correlation with more substantial average improvements, as indicated in Table 4. Notably, a minimal adverse impact of baseline SNOT-22 on NPS changes was identified. It is essential to exercise caution in interpreting this result, given that the previous literature has already reported a lack of association between endoscopic scales and the QoL measured by SNOT-22 [49]. A parallel observation emerges in the analysis of the MLK scale change concerning the baseline image (Table 4). This association may be attributed to the anticipated opacification of the paranasal sinuses and mucosal thickening, stemming not only from polyps but also from the presence of mucosal edema and secretions.

The variability observed in the endoscopic results can be attributed to the baseline differences in the samples studied and to the imprecise delimitation of the different surgical approaches under analysis [26]. Furthermore, postsurgical polypoid recurrences have been reported at a rate exceeding 40% in contrast to 80–85% for edema in the medium and long term [11,19,25,31]. Consequently, the control of endoscopic findings achieved through surgery appears incongruent, and in no instance have authors been able to elucidate the pathophysiological basis for these disparities. Within this context, the expanding understanding of the underlying mechanisms responsible for mucosal inflammation in CRSwNP has prompted the development of more aggressive mucosal techniques, such as reboot surgery [16], and complementary approaches aimed at facilitating improved local healing in both the short and long term [10,50,51]. Fundamentally, these findings underscore the necessity for a precise definition of surgical extent to enable the consistent control of polyp size, edema and mucus in a disease where inflammatory endotypes are gaining prominence. Moreover, as new biologic drugs are beginning to play a role in the treatment of patients who do not achieve CRSwNP control after surgery [52,53], these results underscore the importance of a nuanced understanding of surgical interventions.

Regarding radiological outcomes, our findings align with other studies employing analogous extension techniques, demonstrating a reduction in postoperative LM scores [16,17,48]. Notably, significant distinctions were observed in relation to surgical extension, as illustrated in Figure 2. Nevertheless, upon completion of a multiple linear regression model analysis, it became evident that changes in the radiological scale were exclusively dictated by the baseline values on the LM scale (Table 4). Specifically, these changes remained unaffected by other baseline characteristics or the type of FESS, which differs from other series reporting better outcomes associated with more extended surgeries [16,17]. To better understand these mixed findings, it is important to acknowledge the inherent limitations of the LM scale, which does not differentiate between sinuses that are nearly fully opacified and those that are minimally opacified [54]. Thus, relevant changes in the radiological image derived from the type of surgery may not be evidenced in this scale, with limitations in its graduation. Despite these limitations, our results suggest a substantial impact persisting with E-FESS in terms of radiological findings. However, additional research is required to corroborate our results.

### 4.3. Revision Surgery Rates and Surgical Complications

Finally, the long-term reintervention rates in our sample study parallels with those previously reported by other authors with frequencies of 26% after two years [16], 21% at 5 years [53] or 20% at a mean follow-up of seven years [45]. Better results were observed for the revision rates of the E-FESS group (<10%), as in previous studies ranging from 8 to 23% [10,16,25,38,55]. Furthermore, E-FESS approaches were not associated with a greater number of major postoperative complications compared to L-FESS (*p* = 0.079), which is consistent with previous results reported by Bachert et al. [4], as well as those reported by a large retrospective study that analyzed reinterventions in a large sample of 50,000 patients [56]. These findings lead credibility to the concept of utilizing extended approaches upfront in the surgical management of patients with moderate-to-severe CRSwNP as a reduced number of reinterventions in the long-term is reported.

### 4.4. Limitations and Future Work

This study is limited by its unicentric, non-randomized and retrospective design, which may have led to baseline differences between groups similarly to those observed in other studies [17,19,24,55]. In this study, an analysis of mean change of outcome variables and multiple lineal and logistic regression logistic models has allowed us to statistically control and adjust the outcomes. In addition, patient recruitment was performed in the same period and in a consensual manner among the surgeons of the unit to ensure the inclusion of confirmed moderate-to-severe CRSwNP patients with ESS criteria. Further development of new prospective randomized multicenter studies will allow for larger and homogeneous samples.

On the other hand, the follow-up period was limited to two years. In this line, surgical outcomes have been reported not to change considerably after this period [20,25,48], but with little published evidence, new studies to better understand the effects of surgery in the long term are still needed. Additionally, it must be noted that recent guidelines advise earlier interventions with biological drugs in CRSwNP patients with poor surgical outcomes [1,2,3,5]. This new indication could change the current postsurgical follow-up paradigm, especially in patients with more severe and recalcitrant phenotypes.

Another limitation stems from the lack of consensus in the definition of surgeries. Even though several authors have proposed the definition of some surgical techniques, a common criterion is yet to be reached [26]. Currently, more aggressive extensions over the bony boundaries of the frontal and maxillary sinuses are being introduced for the management of T2 forms of CRSwNP, but the indications for these variants are based solely on expert experience. These conditions are major limitations when comparing surgical groups and the results reported by different studies. In this sense, the need for a classification regarding ESS standards is highlighted, including resection of lamellae, opening of the ostium, extension over the paranasal sinuses and management of the sinonasal mucosa, among others.

## 5. Conclusions

The application of the expanded FESS variant leads to improved quality of life outcomes and the likelihood of achieving responder status for surgery when compared to traditional L-FESS in CRSwNP patients two years after surgery, irrespective of any comorbidity. In addition, E-FESS also promote the further improvement of endoscopic and radiological scales, showing superior management of nasal growth recurrence and edema and decreasing the need for revision surgeries. These findings contribute valuable insights to the evolving landscape of endoscopic sinus surgery. To thoroughly assess these results, further randomized clinical studies, incorporating prospective follow-up and larger sample sizes, are warranted.

## Figures and Tables

**Table 1:** Comprehensive description of the features for limited functional endoscopic sinus surgery (L-FESS) and expanded FESS (E-FESS), considering each of the anatomical structures of the sinonasal cavity (adapted from Martin-Jimenez, D et al. CAAR (2023) [26]).

	L–FESS	E–FESS
**Rationale**	Conservative approach targeting osteomeatal complex disease to allow for proper sinus ventilation, mucociliary clearance and easy topical therapy instillation	To address the affected sinuses (CT images), irrespective of the presence of specific sinus-related symptoms; complete removal of all ethmoidal lamellae can prevent unintended obstruction and facilitate postoperative diagnostics and treatment in the patient
**Objective**	To clear diseased ethmoid clefts and compartments and to re-establish ventilation and drainage of the diseased larger sinus via their physiological routes; damage to the surrounding tissue is minimized	E-FESS is a term used to describe uncinectomy, maxillary antrostomy, total ethmoidectomy, wide sphenoidotomy and a Draf IIA frontal sinusotomy
**Mucosa**	Disease is targeted for removal in key areas of the anterior ethmoid and middle meatus; preservation of as much mucosa as possible	Targeted removal of disease from key areas of the ethmoid and middle meatus
**Uncinectomy**	Performed systematically	Performed systematically
**Ethmoidal bulla**	Once the cell walls are fractured, they are removed	Once the cell walls are fractured, they are removed
**Middle turbinate**	Preservation is preferred	Consider the possibility of medializing the middle turbinate or securing it to the septum through the induction of synechiae
**Vertical plate of the basal lamella of the middle turbinate**	The basal lamella is perforated to enter the posterior ethmoid cells whenever needed	The basal lamella is perforated to enter the posterior ethmoid cells and the opening is enlarged
**Ethmoid bony lamellae**	On demand	Removed systematically
**Middle meatal antrostomy**	On demand	As large as possible
**Maxillary sinus mucosa**	Localized irreversible disease is removed to the periosteum; frequently, apparently irreversible mucosal disease resolves	On demand
**Ethmoid sinus mucosa**	On demand	Removed systematically to the periosteum
**Sphenoidotomy**	On demand	Wide
**Sphenoid sinus mucosa**	Preserved	Preserved unless grossly abnormal
**Frontal sinus opening**	Clearing the frontal recess in most cases of inflammatory processes will also produce healing of the sinus without the need for additionally enlarging the sinus ostium itself	Specified Draf IIA Only Draf III when indicated
**Frontal sinus mucosa**	Preserved	Frontal pathway clearance
**Adjunct procedures**		Canine fossa trephination and frontal minitrephination whenever necessary

**Table 2:** Simple and multiple linear regression effect estimates for study variables in SNOT-22 change.

	Simple Linear Regression Model β, μ_x_ ± SE	Multiple Linear Regression Model Adjusted β, μ_x_ ± SE	95% CI	*p* Value	R^2^
**E-FESS** (**type of surgery**)	**8.2 ± 3.7**	**-**	(**0.9 to 15.5**)	**0.028**	**0.028**
	**-**	12.2 ± 5.0 *^1^	(**2.3 to 22.0**)	**0.016**	**0.396**
**Baseline SNOT-22**	**0.5 ± 0.1**	**-**	(**0.4 to 0.7**)	**<0.001**	**0.233**
	**-**	0.7 ± 0.1 *^1^	(**0.4 to 0.9**)	**<0.001**	**0.396**
**Age**	−0.02 ± 0.1	**-**	(−0.3 to 0.3)	0.917	0.000
	-	0.01 ± 0.2	(−0.4 to 0.4)	0.944	0.396
**Female** (**gender**)	3.3 ± 3.9	**-**	(−4.5 to 11.0)	0.404	0.004
	-	−7.1 ± 5.4	(−17.7 to 3.6)	0.190	0.396
**Asthma**	5.1 ± 3.7	-	(−2.2 to 12.5)	0.170	0.011
	-	−3.3 ± 5.8	(−14.8 to 8.3)	0.577	0.396
**N-ERD**	−2.4 ± 4.6	-	(−11.4 to 6.6)	0.600	0.002
	-	0.3 ± 6.2	(−12.0 to 12.5)	0.966	0.396
**Previous ESS**	−3.6 ± 4.1	-	(−11.6 to 4.4)	0.370	0.005
	-	−9.3 ± 5.5	(−20.3 to 1.6)	0.095	0.396
**Eosinophils in peripheral blood** (**cells/μL**)	5.4 ± 5.5	-	(−5.5 to 16.3)	0.328	0.006
	-	6.4 ± 6.3	(−6.0 to 18.9)	0.306	0.396
**Total IgE** (**UI/L**)	0.01 ± 0.0	-	(0.0 to 0.01)	0.067	0.032
	-	0.01 ± 0.0	(0.0 to 0.01)	0.062	0.396
**Proven allergic sensitization** (**atopy**)	5.6 ± 4.7	-	(−3.7 to 14.8)	0.234	0.011
	-	3.6 ± 5.0	(−6.3 to 13.6)	0.470	0.396
**≥1 cycles of SCS in the pre–surgery year**	3.0 ± 3.8	-	(−4.5 to 10.4)	0.431	0.004
	-	3.6 ± 4.9	(−6.1 to 13.4)	0.458	0.396
**Baseline NPS**	**2.8 ± 1.1**	-	(**0.7 to 5.0**)	**0.009**	**0.040**
	-	1.7 ± 1.7	(−1.7 to 5.2)	0.314	0.396
**Baseline MLK scale**	0.2 ± 0.8	-	(−1.4 to 1.9)	0.793	0.000
	-	−0.3 ± 1.3	(−2.8 to 2.3)	0.844	0.396
**Baseline LM scale**	0.5 ± 0.3	-	(−0.2 to 1.2)	0.142	0.013
	-	0.2 ± 0.5	(−0.8 to 1.3)	0.648	0.396

VIFs were calculated to evaluate multicollinearity in multiple regression models. VIFs were <2.00. *^1^ Multiple linear regression. Model equation with significant predictive factors: Change in SNOT-22= −29.33 + 12.15 × ((1 if E-FESS) or (0 if L-FESS)) + 0.68 × baseline SNOT-22. Abbreviations: μ**_x_** = Arithmetic average; E-FESS = Expanded functional endoscopic sinus surgery; ESS = Endoscopic sinus surgery; IgE = Immunoglobulin E; LM = Lund–Mackay; MLK = Modified Lund–Kennedy; N-ERD = Nonsteroidal anti-inflammatory drug–exacerbated respiratory disease; NPS = Nasal Polyps Score; SCS = Systemic corticosteroids; SE = Standard deviation; SNOT-22 = Sinonasal Outcome Test 22.

**Table 3:** Multiple linear stepwise regression effect estimates for type of surgery in SNOT-22 change.

	Adjusted β (μ_x_ ± SE)	95% CI	*p* Value	R^2^
**E-FESS** (**type of surgery**)	14.8 ± 4.8	(5.5 to 24.1)	**0.002**	0.313
**Baseline SNOT-22**	0.6 ± 0.1	(0.4 to 0.8)	**<0.001**	
**Previous ESS**	−11.2 ± 5.0	(−21.2 to −1.3)	**0.027**	

VIFs were calculated to evaluate multicollinearity in multiple regression models. VIFs were <2.00. Model equation with significant predictive factors: Change in SNOT-22 = −24.7 + 14.8 × ((1 if E-FESS) or (0 if L-FESS)) + 0.62 × (baseline SNOT-22 score) − 11.2 × ((1 if previous ESS) or (0 if no previous ESS)). Abbreviations: μ**_x_** = Arithmetic average; E-FESS = Expanded functional endoscopic sinus surgery; ESS = Endoscopic sinus surgery; SE = Standard deviation; SNOT-22 = Sinonasal Outcome Test 22.

**Table 4:** Multiple linear stepwise regression effect estimates for baseline variables in NPS, MLK scale and LM scale changes.

	Adjusted β (μ_x_ ± SE)	95% CI	*p* Value	R^2^
NPS *				
**Baseline NPS**	0.8 ± 0.1	(0.6 to 1.1)	**<0.001**	0.317
**Baseline SNOT-22**	−0.02 ± 0.01	(−0.04 to 0.0)	**0.046**	
**MLK scale ^t^**				
**Baseline MLK scale**	1.1 ± 0.1	(0.9 to 1.3)	**<0.001**	0.511
**Baseline LM scale**	−0.1 ± 0.04	(−0.2 to −0.02)	**0.013**	
**LM scale ^α^**				
**Baseline LM scale**	0.6 ± 0.1	(0.4 to 0.9)	**<0.001**	0.307

VIFs were calculated to evaluate multicollinearity in multiple regression models. VIFs were <2.00. * Model equation: Change in NPS = 0.8 + 0.8 × (baseline NPS) − 0.02 × (baseline SNOT-22 score). ^t^ Model equation: Change in MLK scale = −0.7 + 1.1 × (baseline MLK scale) − 0.1 × (baseline LM scale). ^α^ Model equation: Change in LM scale = −5.1 + 0.6 × (baseline LM scale). Abbreviations: μ_x_ = Arithmetic average; LM = Lund-Mackay; MLK = Modified Lund-Kennedy; NPS = Nasal polyps score; SE = Standard deviation; SNOT-22 = Sinonasal Outcome Test 22.

## Data Availability

The data presented in this study are available on request from the corresponding author. The data are not publicly available due to ethical reasons.

## Supplementary Materials

The following supporting information can be downloaded at https://www.mdpi.com/article/10.3390/jcm13030866/s1, Table S1: Distribution of demographic variables, previous history of ESS, allergy, and baseline endoscopic, radiological, and quality of life scores in both surgical groups; Table S2: Crude and adjusted odds ratio (OR) and 95% CI for being a responder patient (i.e., achieving MCID = 12 points) and a super-responder patient (i.e., achieving twice the MCID = 24 points) as a function of significant predictor variables; Table S3: Comparison between endoscopic and radiological scores, before and after surgery in the two surgical groups. Reference [27] is cited in the Supplementary Materials.
