# Supplementary material for: Long-Term Clinical Outcomes in Patients with Chronic Rhinosinusitis with Nasal Polyps Associated with Expanded Types of Endoscopic Sinus Surgery
Source: J Clin Med. 2024 Feb 1;13(3):866. doi: 10.3390/jcm13030866 (PMC10856470; doi:10.3390/jcm13030866)
Supplement: Supplementary file 1 [file jcm-13-00866-s001.zip › jcm-2835496-supplementary.pdf]

Supplementary Table S1. Distribution of demographic variables, previous history of ESS, allergy, and baseline endoscopic, radiological, and quality of life scores in both surgical groups.

	**E-FESS (n = 111)**	**L-FESS (n = 163)**	***p v*alue**
**Gender, N (%)**			
**Women**	32 (28.8)	61 (37.4)	0.140*
**Men**	79 (71.2)	102 (62.6)	
**Age, μ_x ± SE**	50.5 ± 13.4	50.0 ± 13.3	0.745[t]
**Asthma, N (%)**	56 (50.5)	79 (48.5)	0.747*
**N-ERD, N (%)**	21 (18.9)	38 (23.3)	0.385*
**Previous ESS, N (%)**	45 (40.5)	45 (27.6)	**0.025***
**Smoking, N (%)**			
**No**	66 (59.5)	100 (61.3)	0.697*
**Ex – smoker**	23 (20.7)	37 (22.7)	
**Smoker**	22 (19.8)	26 (16.0)	
**Eosinophils in peripheral blood > 300/µL, N (%)**	66 (59.5)	107 (65.6)	0.324*
**Total IgE > 150 UI/L, N (%)**	60 (54.1)	84 (51.5)	0.705*
**Proven allergic sensitization (atopy), N (%)**	56 (50.5)	89 (54.6)	0.505*
**≥1 cycle of SCS in the pre–surgery year, N (%)**	55 (49.5)	62 (38.0)	0.059*
**NPS (sum of both nostrils), μ_x ± SE**	5.3 ± 1.7	4.7 ± 1.7	**0.013[t]**
**MLK scale (sum of both nostrils), μ_x ± SE**	8.6 ± 2.0	7.6 ± 2.2	**<0.001[t]**
**Lund–Mackay scale, μ_x ± SE**	16.8 ± 5.1	15.0 ± 5.6	**0.008[t]**
**Baseline SNOT–22, μ_x ± SE**	68.1 ± 21.2	62.1 ± 23.0	0.073[t]

* p-value as determined by chi–square test. [t] p-value as determined by the unpaired T–student test.

Abbreviations: μ_x = Arithmetic average; E-FESS = Expanded functional endoscopic sinus surgery; IgE = Immunoglobulin E; L-FESS = Limited functional endoscopic sinus surgery, MLK = Modified Lund Kennedy; N-ERD = Nonsteroidal anti-inflammatory drug–exacerbated respiratory disease; NPS = Nasal polyp score; SCS = Systemic corticosteroids; SE = Standard deviation; SNOT–22 = Sinonasal Outcome Test 22.

Supplementary Table S2. Crude and adjusted odds ratio (OR) and 95% CI for being a responder patient (i.e., achieving MCID = 12 points) and a super-responder patient (i.e., achieving twice the MCID = 24 points) as a function of significant predictor variables. [*Adapted from*: *Martin-Jimenez, D et al. IFAR, 2024 [27]*]

**Responder patient (MCID = 12 points)**						
	**Crude OR*1**	**95% CI**	***p* value**	**Adjusted OR**	**95% CI**	***p* value**
**E–FESS (type of surgery)**	5.5	(1.3 to 22.8)	**0.019**	6.5*2	(1.7 to 24.8)	**0.006**
**Previous ESS**	0.2	(0.04 to 0.9)	**0.039**	0.2*3	(0.04 to 0.7)	**0.010**
**Super-responder patient (2 x MCID = 24 points)**						
	**Crude OR*1**	**95% CI**	***p* value**	**Adjusted OR**	**95% CI**	***p* value**
**E–FESS (type of surgery)**	3.6	(1.1 to 11.4)	**0.030**	2.9*4	(1.1 to 7.8)	**0.038**
**Previous ESS**	0.4	(0.1 to 1.5)	0.188	0.4*5	(0.1 to 1.0)	**0.014**
**Female**	0.3	(0.1 to 1.1)	0.068	0.3*6	(0.1 to 1.0)	**0.049**
**Baseline SNOT–22**	1.1	(1.0 to 1.1)	**<0.001**	1.1*7	(1.0 to 1.1)	**<0.001**

All baseline variables were included in this predictive model: *gender, age, asthma, N-ERD, previous ESS, smoking, eosinophils in peripheral blood, total IgE, atopy, cycles of systemic steroids, NPS, MLK, Lund-Mackay, baseline SNOT-22, baseline item 21 of SNOT-22.*

*1 Estimated through a crude binary logistic regression. *2 Adjusted by independent significative factors (previous ESS) according to step-by-step multiple logistic model. *3 Adjusted by independent significative factors (type of surgery) according to step-by-step multiple logistic model. *4 Adjusted by independent significative factors (previous ESS, gender and baseline SNOT-22) according to step-by-step multiple logistic model. *5 Adjusted by independent significative factors (type of surgery, gender and baseline SNOT-22) according to step-by-step multiple logistic model. *6 Adjusted by independent significative factors (type of surgery, pervious ESS and preoperative SNOT-22) according to step-by-step multiple logistic model. *7 Adjusted by independent factors (type of surgery, previous ESS and gender) according to step-by-step multiple logistic model.
Abbreviations: E-FESS = Expanded functional endoscopic sinus surgery; SNOT–22 = Sinonasal Outcome Test 22.

Supplementary Table S3. Comparison between endoscopic and radiological scores, before and after surgery in the two surgical groups.

	**E-FESS (n = 111)**			**L-FESS (n = 163)**		
	**Pre–surgery**	**Post–surgery**	***p*-value**	**Pre–surgery**	**Post–surgery**	**p-value**
**NPS (μ_x ± SE)**	5.3 ± 1.7	1.1 ± 1.9	<0.001*	4.7 ± 1.7	1.6 ± 2.3	<0.001*
**MLK scale (μ_x ± SE)**	8.6 ± 2.0	4.1 ± 3.3	<0.001*	7.6 ± 2.2	4.4 ± 3.6	<0.001*
**LM scale (μ_x ± SE)**	16.8 ± 5.1	9.6 ± 4.7	<0.001*	15.0 ± 5.6	11.6 ± 6.3	<0.001*

*p-value as determined by paired T–student test; Me = median, IQR = interquartile range.
